# Developmental contributions to macronutrient selection: a randomized controlled trial in adult survivors of malnutrition

**DOI:** 10.1093/emph/eov030

**Published:** 2016-01-27

**Authors:** Claudia P. Campbell, David Raubenheimer, Asha V. Badaloo, Peter D. Gluckman, Claudia Martinez, Alison Gosby, Stephen J. Simpson, Clive Osmond, Michael S. Boyne, Terrence E. Forrester

**Affiliations:** 1 UWI Solutions for Developing Countries, University of the West Indies, Mona, Kingston, Jamaica; 2 Liggins Institute and National Research Centre for Growth and Development, University of Auckland, Auckland, New Zealand; 3 Charles Perkins Centre and School of Biological Sciences, The University of Sydney, Sydney, NSW, Australia; 4 Faculty of Veterinary Sciences, The University of Sydney, Sydney, NSW, Australia; 5 Tropical Medicine Research Institute, University of the West Indies, Mona, Kingston, Jamaica; 6 MRC Lifecourse Epidemiology Unit, University of Southampton, Southampton, UK

**Keywords:** macronutrient, protein, malnutrition, birthweight, protein leverage

## Abstract

**Background and objectives:** Birthweight differences between kwashiorkor and marasmus suggest that intrauterine factors influence the development of these syndromes of malnutrition and may modulate risk of obesity through dietary intake. We tested the hypotheses that the target protein intake in adulthood is associated with birthweight, and that protein leveraging to maintain this target protein intake would influence energy intake (EI) and body weight in adult survivors of malnutrition.

**Methodology:** Sixty-three adult survivors of marasmus and kwashiorkor could freely compose a diet from foods containing 10, 15 and 25 percentage energy from protein (percentage of energy derived from protein (PEP); Phase 1) for 3 days. Participants were then randomized in Phase 2 (5 days) to diets with PEP fixed at 10%, 15% or 25%.

**Results:** Self-selected PEP was similar in both groups. In the groups combined, selected PEP was 14.7, which differed significantly (*P* < 0.0001) from the null expectation (16.7%) of no selection. Self-selected PEP was inversely related to birthweight, the effect disappearing after adjusting for sex and current body weight. In Phase 2, PEP correlated inversely with EI (*P* = 0.002) and weight change from Phase 1 to 2 (*P* = 0.002). Protein intake increased with increasing PEP, but to a lesser extent than energy increased with decreasing PEP.

**Conclusions and implications:** Macronutrient intakes were not independently related to birthweight or diagnosis. In a free-choice situation (Phase 1), subjects selected a dietary PEP significantly lower than random. Lower PEP diets induce increased energy and decreased protein intake, and are associated with weight gain.

## Teaser

Birthweight in children who had kwashiorkor and marasmus showed no independent effect on the intake of protein and energy in adulthood. However, energy intake and weight gain increase as the percentage of energy derived from dietary protein (PEP) is reduced, hence risk of developing obesity with low PEP diets.

## INTRODUCTION

There is epidemiological and experimental evidence that developmental influences (maternal nutrition, fetal growth, birth size and postnatal nutrition) may modify appetite control and thus the risk of obesity later in life [1, 2]. In animals and humans, birthweight, a marker of *in utero* developmental experience, is associated with macronutrient selection and intake, as well as physical activity, later in life [3–9]. Specifically, offspring who are small for genetic potential have increased caloric intake, decreased physical activity and a tendency to obesity and its comorbidities. Exposure to undernutrition *in utero* as well as in early postnatal life has an especially potent combined developmental influence [10–12].

Children who experience severe undernutrition develop one of two distinct clinical syndromes—oedematous (kwashiorkor and marasmic-kwashiorkor) or non-oedematous (marasmus). We have proposed that those who experienced poor intrauterine nutrition and were born small are more likely to develop the marasmus syndrome when exposed to sustained undernutrition [13, 14]. Marasmic children are better able to sustain supplies of amino acids and lipid to maintain metabolic integrity during acute illness [13, 14], and are probably more susceptible to obesity later in life if exposed to a high-energy environment. On the other hand, children with a developmental history of adequate intrauterine nutrition and normal birthweight develop kwashiorkor when exposed to undernutrition in childhood. When acutely malnourished such children fail to sustain amino acid and lipid supply to their metabolic machinery and thus suffer impaired synthesis of protein and peptides and an energy shortage [15–17]. This metabolic pattern may confer a lower risk of obesity later in life in a high energy environment than the marasmic phenotype.

Although all three macronutrients exert some influence on total energy intake, protein is the most satiating and tightly regulated [18–21]. Because protein appetite control is stronger than that for either fat or carbohydrate, when faced with unbalanced diets with different percentage of energy derived from protein (PEP) humans respond by prioritizing the absolute intake of protein toward a ‘target’ level at the expense of over-ingesting (on low PEP diets) or under-ingesting (on high PEP diets) fats and carbohydrate—an effect that has been called ‘protein leverage’ [19, 22, 23]. According to the Protein Leverage Hypothesis (PLH), the strong regulation of protein intake contributes to the obesity epidemic during nutrition transition when PEP is diluted by cheap, widely available fat and carbohydrate [19, 22–25]. A corollary of protein leverage is that individuals with a high protein target will be more susceptible to energy over-consumption and thus obesity than individuals with a low protein target, because for a given degree of dietary protein dilution meeting a higher protein target will necessitate a greater over-consumption of fat and carbohydrate [22, 25].

We hypothesized that the protein target is related to severe acute malnutrition (SAM) phenotype and birthweight in survivors of SAM. In addition, we hypothesized that the magnitude of the change in total energy intake that occurs with a change in percent dietary protein energy (protein leveraging) to maintain protein intakes at protein target levels would be influenced by *in utero* and postnatal developmental experiences and thus to birthweight and SAM phenotype.

## METHODS

### Study participants

On the basis of the study by Gosby birthweight [18], using a test at the 5% level and an estimated sample size of 20 participants per protein group, we have 80% power to detect a difference in the daily energy consumption of 150 kcal.

Inclusion criteria were males and females, aged 17–50 years and body mass index (BMI) 18–41 kg.m^2^. Participants were excluded from the study if they were diabetic, hypertensive, pregnant, or currently taking appetite altering medication. In total, 63 participants agreed to participate and were recruited between June 2009 and June 2012 (see Supplementary Fig. S3). Subjects provided written informed consent. The study was approved by the Faculty of Medical Sciences Ethics Committee, University of the West Indies. All participants completed the 9-day study period by June 2012 and were included in the final analysis.

Study subjects were recruited from among individuals who had experienced SAM in childhood and who had been rehabilitated on the metabolic ward of the Tropical Metabolism Research Unit (TMRU), University of the West Indies, Kingston, Jamaica. We reviewed the admission records for 1336 patients who had been admitted with SAM between 1963 and 1993. These patients were referred from clinics all over Jamaica as TMRU is the only dedicated nutritional rehabilitation center on the island. For each patient, we extracted from the records clinical (age, gender, presence of edema), anthropometric (weight and length at admission) and survival data as well as recalled birthweight. Birthweights were recalled by the mother at the time of admission. This has been shown to be highly correlated with recorded birthweight [26]. During hospital admission 27 males and 20 females died (4.1%). Survival was not associated with birthweight, nor did the difference between birthweights of patients with marasmus and kwashiorkor differ according to whether they died during hospital admission or not [12]. Using the last known address and name of the parent, we traced 729 individuals in the community. Of these, 312 were available for recruitment, and a further 163 were unavailable to the study as a result of refusal (14), migration (53), illness (18), pregnancy (3) or death (75). The remaining 688 members of the cohort have not been traced.

### Study design

All subjects were seen in single-sex pairs and stayed in a dedicated metabolic suite for nine consecutive days. Subjects arrived for the assigned study period with a completed 3-day food diary, for which they were asked to record their intake on two week days and on a week-end day. Participants were weighed daily.

Measurements were conducted in two phases. During Phase 1 (Days 1–3—choice experiment) they ate freely at each meal-time from menus comprising a combination of foods containing different percentages of energy as protein (PEP), set at 10%, 15% or 25% [26]. The aim of this phase was to establish the pattern of macronutrient selection in a situation where subjects could freely compose a diet by combining foods varying from 10% to 25% PEP. During Phase 2 (Days 4–8), pairs were randomly allocated to one of three groups each of which received menus comprised only of foods that contained 10%, 15% or 25% PEP (10%; *n* = 22, 15%; *n* = 20 or 25%; *n* = 21). In this phase, we aimed to test the extent to which PEP leveraged the intake of non-protein energy when subjects were confined to diets with PEP ranging from relatively low level (10%) to high (25%) PEP. Participants were taken for a 1-h supervised walk each day at 4 p.m.

### Study diet

Before the experiment started each individual was randomized to a PEP diet (10%, 15% or 25%) by a statistician who was blinded to diagnosis and nutritional status. For each 9-day trial (comprising Phase 1 and Phase 2), two individuals (one marasmus; one kwashiorkor) were selected from those previously allocated to each of the diet treatment lists, so that two individuals (one marasmus; one kwashiorkor) participated in each 9-day repeat of the experiment. These persons were then contacted, informed about the objectives, methods, risks and benefits of the study and invited to participate. This pattern was repeated throughout the duration of the trial.

The design, manipulation and taste testing of the foods used are presented in detail elsewhere [27]. Briefly, 31 local recipes of 10 sweet and 21 savory foods were selected. Each was modified into three recipes containing 10%, 15% or 25% energy as protein through the addition of food ingredients, a protein mix and/or maltodextrin (Ross Nutrition). Carbohydrate was adjusted to be 60%, 55% or 45% energy and dietary fat was kept constant at 30%. Energy density (kJ/g) was held similar among the 10%, 15% and 25% PEP versions of each dish/recipe, but could differ among the different types of dishes. Once designed, the PEP versions of each food/recipe were taste tested for the ability to determine the protein concentration of any dish due to appearance, smell or texture as well as for pleasantness [27]. If taste testers were able to detect any difference, the recipes were adjusted while maintaining their assigned macronutrient content and retested until no difference was detected.

Up to 11 foods were provided on each day during the 8-day period, giving participants both variety and choice at all times (see Supplementary Table S1). During the first 3 days (Phase 1), three menu items along with fruit, tea and vegetable salad were offered at breakfast, lunch and dinner to all the participants. These three menu items at each of these meal times included foods containing 10, 15 or 25 PEP. If all three menu items were eaten equally (i.e. no discrimination), this would provide a diet with 16.7 PEP, whereas disproportionate intake of the 10, 15 or 25 PEP foods would result in selected diet of lower or higher PEP, respectively. From Days 4–8 (Phase 2), these same daily food types were repeated every 3 days but the foods all contained 10 PEP in one group, 15 PEP in the second group and 25 PEP in the third group.

Breakfast was provided at 8 a.m., lunch at 12:30 p.m. and dinner at 6 p.m. During both phases, snack items shown in Supplementary Table 1 were made freely available at all times. Participants had free access to any baked products that were first served at a meal and not completely consumed at that meal. The foods were served in weighed quantities in tared containers. Plates were of a single design and neutral (white) color. The same size, style and color plates were used for the 10, 15 and 25 PEP version of each food. Participants were offered optional foods including 100 g fruit salad, and decaffeinated tea (8 oz) sweetened with a fixed amount (22 g) of brown sugar with breakfast, and 100 g vegetable salad with lunch and dinner.

**Table 1.** Characteristics of the cohort

Measurement	SAM phenotype			
	Kwashiorkor		Marasmus	
	Male (*n* = 18)	Female (*n* = 15)	Male (*n* = 14)	Female (*n* = 16)
Measurements recorded at admission with SAM				
Birthweight (g)	3180 (787)	3021 (666)	2894 (724)	1985 (672)
Age (months)	10.1 (4.9)	13.2 (4.3)	12.0 (5.7)	11.0 (5.9)
Height for age (%)	87.7 (4.1)	90.7 (5.5)	85.0 (4.5)	84.7 (7.0)
Weight for age (%)	62.3 (8.5)	66.2 (10.2)	50.5 (5.4)	50.3 (9.7)
Weight for height (%)	85.1 (8.3)	82.7 (13.9)	73.2 (5.2)	74.9 (7.1)
Measurements recorded in adult life				
Age (years)	27.0 (6.2)	28.0 (9.1)	27.3 (6.9)	24.9 (5.1)
Height (cm)	173.6 (8.6)	160.4 (9.3)	170.2 (4.5)	160.9 (7.4)
Weight (kg)	69.4 (11.2)	63.4 (15.1)	60.6 (10.2)	59.5 (16.8)
Body mass index (kg/m**^2^**)	23.1 (3.7)	24.9 (6.8)	20.8 (2.8)	22.7 (5.3)

Values are given as mean (SD). SD, standard deviation.

### Assessment of energy and macronutrient intake

The primary outcome measures were energy and macronutrient intake. The amount eaten was determined by weighing to the nearest gram using an electronic balance (OHAUS Corporation, Pine Brook New Jersey) each food item before consumption, then weighing any of the item that was not eaten. A 3-day food diary completed prior to the 9-day test period was analyzed for total energy, protein, carbohydrate and fat content using the NUTRITIONIST Five (version 2.3, 2000, First Data Bank, San Bruno, CA) software.

### Body weight measurements

A secondary outcome measure was body weight. The weight of the subjects without shoes and in light clothing was measured daily to the nearest 0.1 kg using a Seca balance (Vogel & Halke, Hamburg). Height was measured to the nearest 0.1 cm using a stadiometer (Invicta, London, UK). Weight gain was calculated from Days 1–4 in Phase 1 and from Days 4–8 in Phase 2.

### Statistical analysis

Macronutrient intake in Phase 1 was used to compare the protein target of the M and K groups. We tested for protein leveraging by comparing energy intake in Phase 2 between the 10, 15 and 25 PEP treatment groups, rather than comparing for each subject intake in Phase 1 (target intake) with intake in Phase 2 (intake of fixed PEP). The reason for this is that the two phases of the experiment inevitably differed in important respects over-and-above the experimental manipulation (macronutrient selection vs no-choice, respectively), which were not possible to control. For example, the initial novelty for subjects of being provided with free access to diverse foods in Phase 1 would no longer apply in Phase 2. Further, subjects entered Phase 2 having spent the prior 3 days eating experimental diets *ad libitum* whereas they had eaten their usual diets in the days prior to entering into Phase 1. Of particular note, is the observation that subjects ate more during the first phase of the experiment compared with intake prior to the study, and gained weight (see Results), suggesting that they might not have been in metabolic equilibrium at the point of entering the study, although neither can we rule out the possibility that increased intakes were caused by a novelty effect. We used multiple linear regression analysis to study how the protein target and protein leveraging were associated with sex, birthweight, SAM phenotype and SAM admission measurements, current age, weight and height and the protein energy ratio of the diet in Phase 2. Sex, current age and weight were included in each model. Adjustment was done for the effect of clustering. To compare the PEP of the selected diet in Phase 1 with a null value of 16.7 (equal intakes of 10, 15 and 25 PEP foods), for each subject observed PEP was subtracted from 16.7 and the difference variable tested against 0 using a one-sample *t*-test.

## RESULTS

### Design and subject characteristics

Table 1 shows subjects’ anthropometry measurements both as infants at admission with SAM and at the start of the feeding trial when they were adults. Survivors of kwashiorkor were heavier at birth than survivors of marasmus (mean difference = 665 g, 95% confidence interval (CI) 252–1078, *P* = 0.002). There was no significant difference in height, weight or BMI between the adult survivors of kwashiorkor and marasmus (*P* > 0.1).

### Pre-study habitual diet

The PEP of the pre-study habitual diet was estimated to be 15 ± 3.1. Across all the participants, reported energy intake was 1904 ± 884 kcal/day (31 ± 16 kcal/kg/day). Mean energy and protein intake in males were 1976 ± 881 kcal/day and 74 ± 36 g/day, respectively; whereas mean energy and protein intake in females were 1874 ± 906 kcal/day and 71 ± 37 g/day.

#### Phase 1: choice experiment

**Figure 1.**
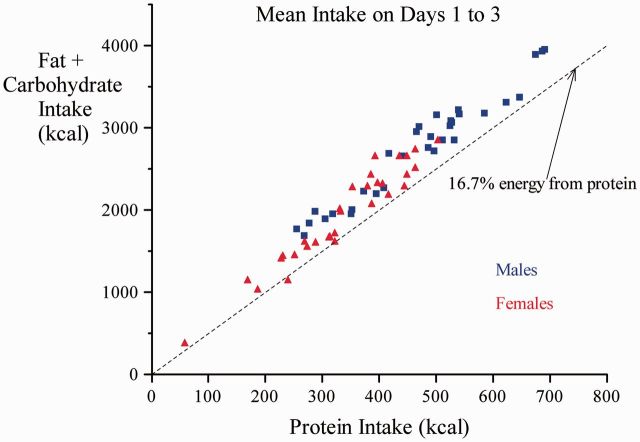
Self-selected daily protein vs non-protein energy (carbohydrate and fat) intake during Phase 1. The mean total intake across all 3 days was 14.7% protein, which differed significantly (*P* < 0.0001) from the null expectation (16.7%) of no selection among the 10, 15 and 25 PEP foods provided at mealtimes whether sexes were combined or tested separately

**Figure 2.**
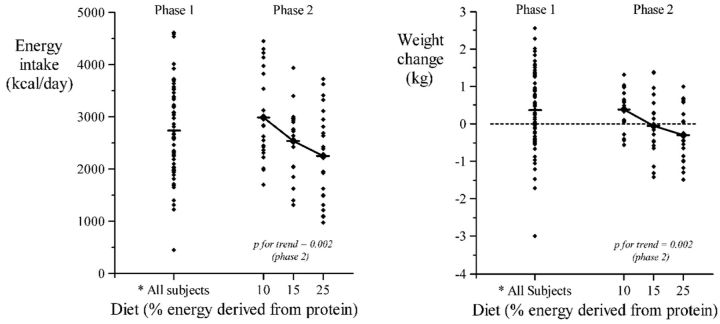
Energy intake and weight change during Phase 1 and according to diet assigned in Phase 2*Phase1: all subjects self-selected a diet from a combination of foods containing 10%, 15% and 25% of energy as protein. The mean intake was 14.7%

The average energy intake by the participants during the study was 2727.93 ± 13.24 kcal/day and 43.64 ± 13.24 kcal/day/kg which was higher than their habitual intake (*P* < 0.0010). Energy derived from protein for all subjects was 402 ± 114 kcal/day and 6.4 ±1.9 kcal/day/kg. Men consumed significantly more absolute protein and protein per kilogram body weight expressed as protein energy (468 ± 126 kcal/day, 7.3 ± 2.1 kcal/day/kg) than women (337 ± 102 kcal/day, 5.6 ± 1.7 kcal/day/kg). Multiple regression analysis indicated that protein intake was 120 kcal/day higher in men than women (95% CI 65–174, *P* < 0.001) and 2.3 kcal/day greater per kilogram of weight (0.1–4.4, *P* = 0.04), but was not further associated with subjects’ current age or anthropometry (height, body mass index, all *P* values > 0.19). Protein intake was not significantly greater in kwashiorkor survivors than in marasmus survivors (15 kcal/day, −41 to 71, *P* = 0.60) nor was there a significant difference with birthweight (−39 to 41, *P* = 0.97) between these groups. In achieving their higher protein target men consumed more total energy, but the PEP did not differ from females (men: 14.66 ± 0.86 and women 14.85± 0.78). The latter was not different from the habitual PEP for the males (14.75 ± 2.3) and females (15.1. ± 3.6), but did differ significantly from the null value of 16.7, whether sexes were combined or tested separately (*P* < 0.0001; Fig. 1). The higher total energy intake in males was associated with higher current body weight, but was not associated with age, other measures of anthropometry (height, BMI) (not shown), the SAM phenotype, or the subsequent diet allocation (see Fig. 2). Bivariate analysis shows a significant effect of birthweight on PEP; PEP in the diets consumed by participants fell by 0.36% per kg birthweight (0.04–0.69, *P* = 0.03); the effect was lost after controlling for age, sex and weight (Table 3). Further adjustment for clustering did not significantly change these outcomes.

##### Weight change

Mean weight gain in the participants was 0.37 ± 1.02 kg/day (Fig. 2). Table 2 shows mean energy intake and weight change in Phase 1 according to sex and SAM phenotype. Table 3 shows the results of regression models for weight change. Mean weight change was 1.05 kg higher in men than women, but was not related to age, anthropometry, SAM phenotype, birthweight or subsequent diet allocation. As expected, weight change was strongly associated with energy intake; with every 1000 kcal extra consumed per day during this 3-day period predicting an increase in weight of 0.66 kg (95% CI 0.39–0.94, *P* < 0.001). Similar values applied to men and women, and in survivors of marasmus and kwashiorkor. Adjustment for energy intake reduced the difference in mean weight gain between men and women from 1.05 to 0.40 kg (−0.03 to 0.84, *P* = 0.07).

**Table 2.** Energy intake and weight change according to SAM phenotype, sex, study phase and assigned diet

Measurement	SAM phenotype			
	Kwashiorkor		Marasmus	
	Male	Female	Male	Female
Phase 1				
Number	18	15	14	16
Energy intake (kcal/day)	3304 (779)	2368 (720)	3008 (734)	2173 (658)
Energy intake ((kcal/day)/kg)	48.8 (14.1)	37.6 (8.9)	50.1 (11.6)	37.7 (12.8)
Weight change (kg)	0.79 (0.86)	−0.15 (0.75)	1.02 (1.26)	−0.16 (0.65)
Phase 2				
Number	18	15	14	16
Energy intake (kcal/day)	3135 (747)	2124 (690)	2997 (732)	2069 (693)
Energy intake ((kcal/day)/kg)	46.1 (12.7)	33.2 (6.7)	49.8 (10.9)	36.3 (13.5)
Weight change (kg)	0.02 (0.93)	−0.12 (0.54)	0.16 (0.67)	0.04 (0.73)
Phase 2, 10% protein				
Number	6	5	5	6
Energy intake (kcal/day)	3458 (900)	2704 (247)	3547 (871)	2278 (467)
Phase 2, 15% protein				
Number	5	5	5	5
Energy intake (kcal/day)	2882 (683)	2131 (666)	2770 (403)	2329 (733)
Phase 2, 25% protein				
Number	7	5	4	5
Energy intake (kcal/day)	3040 (654)	1537(556)	2997 (732)	1559 (720)

Values are given as mean (SD). SD, standard deviation.

#### Phase 2: no choice experiment

Supplementary Table S2 shows the allocation of the 63 subjects to the study diets with PEP of 10%, 15% or 25% according to sex and SAM phenotype.

##### Energy intake

Table 2 shows total energy intake in Phase 2 according to sex and SAM phenotype. The regression models (Table 3) show that energy intake was higher in men than women and increased with current body weight, but was not associated with age, other measures of anthropometry (not shown), SAM phenotype or birthweight. Importantly, there was a strong association with allocated diet (*P* for gradient across the three groups = 0.002, see Fig. 2) where total energy intake was inversely related to percent dietary protein. The gradient was similar in men and women, and in survivors of marasmus and kwashiorkor (*P* for interaction = 0.6 in both cases).

**Table 3.** Regression models in which protein, carbohydrate + fat and energy intake, and weight change, in Phases 1 and 2, are explored in relation to the subjects’ age, sex, weight, SAM phenotype, birthweight and assigned diet

		Protein intake (kcal/day)		Carbohydrate + fat intake (kcal/day)		Total energy intake (kcal/day)		Weight change (g)	
		Phase1	Phase2	Phase1	Phase2	Phase1	Phase2	Phase1	Phase2
Model 1									
Age (years)	*B*, SE(*B*)	3.3. 2.1	6.8, 2.9	15, 11	15, 12	18, 13	22, 12	26, 18	19, 15
	*P*	0.1	0.02	0.2	0.2	0.2	0.08	0.1	0.2
Sex (M = 1, F = 0)	*B*, SE(*B*)	120, 27	170, 37	709, 145	714, 155	821, 170	880, 158	1052,225	91, 186
	*P*	<0.001	<0.001	<0.001	<0.001	<0.001	<0.001	<0.001	0.6
Weight (kg)	*B*, SE(*B*)	2.3, 1.1	0.6, 1.4	15, 6	20, 6	17, 7	20, 6	−7, 9	4, 7
	*P*	0.04	0.7	0.009	0.002	0.01	0.002	0.4	0.6
Model 2 = Model 1 +									
Diagnosis (M = 1, K = 0)	*B*, SE(*B*)	−15, 28	−23, 38	—102,148	94, 159	—116,174	64, 162	110, 231	212, 190
	*P*	0.6	0.5	0.5	0.6	0.5	0.7	0.6	0.3
Model 3 = Model 1 +									
Birthweight (kg)	*B*, SE(*B*)	−1, 20	−26, 23	62, 104	21, 133	62,122	−6, 113	15, 168	−66, 135
	*P*	1.0	0.3	0.6	0.9	0.6	1.0	0.9	0.6
Model 4 = Model 1 +									
Diet 10% (Y = 1, N = 0)	*B*, SE(*B*)	49, 34	−247, 33	296, 178	893, 155	345, 209	617, 181	525, 275	678, 217
	*P*	0.2	<0.001	0.1	<0.001	0.1	0.001	0.06	0.003
Diet 15% (Y = 1, N = 0)	*B*, SE(*B*)	18, 34	−147, 33	53, 177	498, 154	73, 207	333, 180	276, 274	279, 215
	*P*	0.6	<0.001	0.8	0.002	0.7	0.07	0.3	0.2

SE, standard error.

##### Weight change

Table 2 shows weight change in Phase 2 according to sex and SAM phenotype. Table 3 shows the results of regression models for weight change. Weight change was not associated with age, sex, weight, other measures of anthropometry, SAM phenotype or birthweight. There was a strong association with allocated diet (*P* for gradient across the three groups = 0.002, see Fig. 2). The gradient was similar in men and women, and in survivors of marasmus and kwashiorkor (*P* for interaction = 0.8 and 0.5, respectively). However, weight change was strongly linked to energy intake. Every 1000 extra kcal consumed per day during Phase 2 was associated with an increase in weight of 0.70 kg in men (0.36–1.03, *P* < 0.001) and of 0.74 kg in women (0.45–1.04, *P* < 0.001). Furthermore, the gradient across the allocated diet groups was removed by controlling for energy intake (after adjustment, *P* for gradient across the three groups = 0.3). Further adjustment for clustering did not significantly change these outcomes.

## DISCUSSION

### Phase 1

We expected birthweight and the significant differences in birthweight with childhood SAM syndrome (M & K) to influence primarily protein and, via protein leveraging, energy intake on PEP-imbalanced diet in adult survivors of SAM. Although there was a significant difference in birthweight between diagnostic groups, there was no independent significant effect of childhood diagnosis of SAM (M & K) or birthweight on intake of protein and total energy, suggesting no effect on appetite control and satiety regulation in the participants.

Survivors of kwashiorkor were heavier at birth than survivors of marasmus (mean difference = 665 g). This difference is greater than shown by Forrester *et al.* (2012) [13], possibly because the present study (*n* = 63) is a small subset of the larger study group (*n* = 1336) and there is some overlap in birthweight between diagnoses as has been shown in the previous study. Bivariate analysis showed a significant effect of birthweight on PEP, but the effect was lost after controlling for age, sex and weight. This could be because the effect of birthweight is acting more strongly through its interaction with sex and body weight, because both sex and body weight were significantly related to protein intake and these are associated with birthweight.

In the free-choice stage of our experiment (Phase 1), men gained weight whereas the women lost weight during the same period. This may be attributed to the males consuming significantly more energy during the study (3156 ± 757 kcal/day) compared with their habitual intake (1976 ± 881 kcal/day^)^. The women also consumed more energy but to a lesser extent (1874 ± 906 vs 2271 ± 689 kcal/day). This difference in intake and weight gain might also reflect different psychology affecting appetite and body image between the sexes.

Overall, based on the differences between intake and weight before and after the study, the question arises as to whether amounts of protein eaten during this phase of the experiment represent the normal protein target in a steady state. The protein target might be expected to be close to the normal protein requirement, but the intake in this phase (1.8 g/kg/day) is about twice the protein requirement in adults cited by the WHO (0.83 g/kg/day). On the other hand, recent evidence suggests that human protein requirements have been significantly under-estimated, with the true population safe intake for adult men being 1.2 g/kg/day [28]. A recent analysis of compiled data from published experiments on human macronutrient regulation suggests that this is very close to the regulated protein intakes of subjects on a diet of 15% PEP [21]. Significantly, PEP of 15 is similar to the diet of 14.76 ± 0.82 selected during free-choice in the present study, and to the habitual diets of the subjects in our study. It thus seems likely that the absolute protein intakes observed in Phase 1 of our study are close to expected regulated intakes for a diet of approximately 15 PEP, and not greatly in excess of requirements.

The effect of birthweight on food and macronutrient intake has been shown in a number of studies. Two epidemiological studies in a cohort exposed to the Dutch famine during gestation observed that such an exposure was associated with an increased intake of fat in later life [6, 7]. In a more recent study of participants in the Helsinki birth cohort, it was reported that small size at birth was associated with lower intake of carbohydrates and higher intake of fats [8]. However, in that study a stronger association was observed between ponderal index at birth than between birthweight and adult life macronutrient intake. Moreover, in both the Dutch famine studies [6, 7] and the Helsinki birth cohort study [8] the habitual fat intake of the study population was much greater (34 E% and 36 E%) and carbohydrate intake much lower (44 E%) compared with our study. In addition, it has been proposed that aging may alter food intake and food preferences, which could explain different findings between our study in which the age range was 17–56 years and the other studies which involved older adults. The lack of an effect of birthweight on intake in the present study could be related to metabolic differences associated with exposure to SAM as well as social factors influencing intake and nutritional status.

### Phase 2

In this phase of the study, we hypothesized that protein leveraging would be influenced by birthweight and SAM type. We expected to demonstrate protein leverage by an increase in energy intake as PEP decreases in order to satisfy the target protein as determined in Phase 1. Similar to Phase 1, energy intake was influenced by sex and weight but not by SAM phenotype or birthweight and we therefore combined the entire sample for analysis. A limitation is that at the start of Phase 1, the participants might not have been in a stable metabolic state as seen from the difference in dietary intake prior to the study and the weight gain during the study. This is a confounder that limits the testing of our hypothesis using Phase 1 target intake as the reference against which to compare leveraged energy intakes in Phase 2. We could, nonetheless, test for protein leverage by comparing energy intakes between dietary treatments within Phase 2.

As predicted, energy intake was inversely proportional to dietary PEP, rising progressively as PEP fell from 25% to 10%. This finding is in agreement with a meta-analysis of 38 *ad libitum* dietary trials which also reported a strong negative relationship between energy intake and percent dietary protein, most notably across the range from 10% to 25% protein [19]. In the present study, there was also an increase in weight of 0.72 kg for every 1000 kcal/day increase in energy consumed. This result supports the hypothesis that a nutritional environment which encourages dilution of dietary protein with fat and/or carbohydrate can promote increased total energy intake and thus increase the risk of developing obesity. Many sources of such dilution exist in environments undergoing nutritional transition, where fat and carbohydrate are cheaper than protein [29]; there is an increased reliance on processed foods which are often higher in fat and refined carbohydrate than unprocessed foods [24].

Gosby *et al.* [18] also tested the PLH using macro-nutritionally disguised diets as in the present study, and found that lowering the percent protein of the diet from 15% to 10% resulted in higher total energy intake. They suggested that increased energy intake was not sufficient to maintain protein intake constant, indicating that protein leverage was incomplete. In contrast to our study, Gosby *et al.* [18] found that increasing protein from 15% to 25% did not alter energy intake. Differences in the design between the present study and that of Gosby *et al.* [18] were the number of subjects (*n* = 63 compared with *n* = 22), characteristics of the subjects (exposure vs no exposure to childhood malnutrition) and duration of the non-choice experimental periods (one period for 5 days compared with three non-consecutive periods for 4 days). Another recent study using non-disguised diets found reduced energy intake on 25% protein diet but no evidence of increased energy intake on a very low (5%) protein diet [30]. At very low levels such as with 5% PEP, which is approximately equivalent to protein levels in white bread and lower than habitually eaten by any human society with food sufficiency, there can be reduced appetite in association with severe deficiencies in protein.

In addition to total energy intake, PEP had a significant positive effect on protein intake, albeit to a lesser extent than PEP influenced energy intake, as previously observed by Gosby *et al.* [18] and in a secondary analysis of published trials from the literature [21]. This pattern reflects the fact that the intake of non-protein energy is regulated to some extent (i.e. compensation for low non-protein energy on high PEP diets results in over-consumption of protein), but is outweighed by stronger regulation of protein intake [21]. In light of the evidence linking high protein intakes with poor metabolic health [31, 32], this has significant implications for high-protein weight loss diets, and considered together with the excess energy intake observed on low PEP diets underscores the importance of dietary macronutrient balance [21]

## LIMITATIONS

A potential limitation of this study is that subjects selected a higher total energy intake in Phase 1 compared with reported habitual energy intakes, and we cannot thus be certain of the extent to which this represented chronic energy shortage in their normal environment or the novelty of the experimental environment. This does not, however, affect our demonstration of protein leverage in Phase 2, which was indicated by the negative relationship between energy intakes and diets with fixed PEP of 10%, 15% or 25%. Plausibly, it could however be relevant to the question of whether our experiment demonstrated bidirectional protein leverage (i.e. effective on diets with both higher and lower PEP than the target PEP), or unidirectional (i.e. only on high or low PEP diets). For example, if the observed self-selected PEP in Phase 1 (14.7%) is representative of habitual PEP, then our results suggest bi-directional protein leverage, because in Phase 2 subjects on 10% and 25% PEP diets ate more and less energy, respectively, than 15% PEP diets. However, if the selected PEP in Phase 1 was an artefact due to subjects having entered the experiment in a state of metabolic imbalance, then the experiment might only have provided evidence for uni-directional leverage. If, for example, the true target PEP was 25%, then we would need to have included a treatment with PEP higher than 25% to conclude that protein leverage was effective on diets with surplus P, and if the true target PEP was 10% we would need to test diets with PEP <10% to demonstrate leverage on low-P diets. However, it seems highly likely that the PEP of the selected diet (14.7%) was not affected by the prior circumstances of subjects, given that it did not differ significantly from the PEP of the reported habitual diet. Further, globally there are very few human societies with food sufficiency that eat a diet outside of the 10–25% PEP range. Another limitation is that body weight was measured by three persons, one of whom was not blinded to the dietary allocations or patient diagnosis. However training and certification in all measurements were provided at the start of the study, and inter observer reliability was assessed every 4 months.

## CONCLUSIONS

There was no independent significant effect of childhood diagnosis of SAM (M & K) on the intake of protein and total energy, suggesting no effect on appetite control and satiety regulation in the participants. However low birthweight was associated with higher protein targeting, although the effect of birthweight may be mediated through body weight. The inverse relationship between EI and PEP in Phase 2 demonstrates protein leverage, whereas the increase in protein intake with increasing PEP suggests that the strength of protein regulation did not entirely override regulation of carbohydrate and fat intake. Our results are strongly suggestive of bi-directional protein leverage—i.e. increased energy intake on diets with low PEP relative to the target as well as decreased energy intake on diets with high PEP relative to the target.

It would be interesting to explore whether protein target varies across population with different intergenerational nutritional plane is a key question whose answer would illuminate obesity epidemics in population before and during the nutritional transition. However, as food insecurity might have a strong effect on appetite, it will be important to design experiments that assure metabolic stability whereas protein and protein leverage are being assessed. Similarly, the impact of intrauterine nutritional exposures free of postnatal malnutrition needs to be elucidated; this has relevance to prematurity and intrauterine growth retardation especially a more complete understanding of the impact of feed composition on appetite later in life.

## SUPPLEMENTARY DATA

Supplementary data is available at *EMPH* online.

## FUNDING

This project was funded by HRC International Investment Opportunities (Grant no. 09/052) “Developmental adaptation to an obesogenic environment”. Project ID No. 0003622389.

## Supplementary Material

Supplementary Data
