# Supplementary material for: Developmental contributions to macronutrient selection: a randomized controlled trial in adult survivors of malnutrition
Source: Evol Med Public Health. 2016 Jan 27;2016(1):158–69. doi: 10.1093/emph/eov030 (PMC4871598; doi:10.1093/emph/eov030)
Supplement: Supplementary Data [file eov030_Supp.zip › Supplemental Data_Menu + Subject allocation.pdf]

Table 4.

Menu items (repeated every 3 days) for the 8-day ad libitum diets

MEAL	STUDY DAY 1	STUDY DAY 2	STUDY DAY 3
**Breakfast 8-9am**	Cheesy cornmeal muffin<br>Apple crumble muffin<br>Johnny cakes<br>Tea (sugar)<br>Fruit salad (apple+ watermelon)	Porridge (peanut)<br>Banana carrot muffin<br>Cheese scones<br>Tea (sugar)<br>Fruit salad (apple+ watermelon)	Banana bread<br>Banana Porridge<br>Savoury muffins<br>Tea (sugar)<br>Fruit salad (apple + watermelon)
**Lunch 12:30pm**	Red peas soup<br>Curried potatoes and chickpeas<br>Tuna Bake<br>Tossed salad (lettuce, tomato, carrot)	Salsa chili<br>Cheesy peppered cornbread<br>Mackerel fried rice<br>Tossed salad (lettuce, tomato, carrot)	Chicken chow mien<br>Potato cheese casserole<br>Rice and peas<br>Tossed salad (lettuce, tomato, carrot)
**Dinner 6pm**	Macaroni and cheese<br>Seasoned rice<br>Sweet potato coconut pone<br>Tossed salad (lettuce, tomato, carrot)	Lasagna (beef/chicken)<br>Bean stew<br>Bread pudding<br>Tossed salad (lettuce, tomato, carrot)	Meatloaf (beef/chicken)<br>Pelau<br>Sweet potato coconut pone<br>Salad
**Snack**	Orange poppy seed cake<br>Cornmeal pudding	Carrot cake<br>Coconut custard	Bread pudding<br>Apple crumble cake

Each food item, except for tea, fruit and vegetable salads were prepared as three versions containing 10%, 15% and 25% protein energy.

Table 5. Experimental Design. Allocation of the 63 subjects.

Diagnosis	Gender					
	Male			Female		
	Protein (%), days 4 to 8			Protein (%), days 4 to 8		
	10	15	25	10	15	25
**Marasmus**	5	5	4	6	5	5
**Kwashiorkor**	6	5	7	5	5	5
