# Supplementary material for: Developmental contributions to macronutrient selection: a randomized controlled trial in adult survivors of malnutrition
Source: Evol Med Public Health. 2016 Jan 27;2016(1):158–69. doi: 10.1093/emph/eov030 (PMC4871598; doi:10.1093/emph/eov030)
Supplement: Supplementary Data [file eov030_Supp.zip › Supplemental Data_The association of sex_age_diagnosis_body weight_protein group with energy and protein intake.pdf]

Table 6: The association of sex, age, diagnosis (kwashiorkor/marasmus), body weight and protein group (10%, 15%, 25%) with energy intake in the two study phases.

Analyses shown with and without adjustment for clustering

Regression terms	Mean daily energy intake (kcal/day)	
	Unadjusted for clustering	Adjusted for clustering
	**B ± se; P**	**B ± se; P**
**Phase 1 (days 1-3)**		
Constant	1832 ± 514, 0.001	2361 ± 489, <0.001
Female sex (1=Y, 0=N)	-833 ± 169; <0.001	-828 ± 208; <0.001
Age (years)	18.4 ± 13.3; 0.17	9.1 ± 11.4 ; 0.43
Marasmus (1=Y, 0=N)	-153 ± 174; 0.38	-246 ± 144; 0.10
Body weight (kg)	12.8 ± 7.1; 0.07	8.3 ± 6.1; 0.18
Diet 10% (1=Y, 0=N)	284 ± 211; 0.18	381 ± 257; 0.15
Diet 25% (1=Y, 0=N)	-84 ± 208; 0.69	-54 ± 258; 0.84
**Phase 2 (days 4-8)**		
Constant	1466 ± 450, 0.002	1648 ± 431, <0.001
Female sex (1=Y, 0=N)	-910 ± 148; <0.001	-891 ± 179; <0.001
Age (years)	23.7 ± 11.6; 0.05	17.7 ± 10.2; 0.09
Marasmus (1=Y, 0=N)	1 ± 152; 1.0	-47 ± 129; 0.72
Body weight (kg)	15.0 ± 6.2; 0.02	14.6 ± 5.4; 0.01
Diet 10% (1=Y, 0=N)	284 ± 185; 0.13	321 ± 221; 0.16
Diet 25% (1=Y, 0=N)	-333 ± 182; 0.07	-325 ± 221; 0.15

B = regression coefficient; se = standard error; P = P-value.

Table 7: The association of sex, age, diagnosis (kwashiorkor/marasmus), body weight and protein group (10%, 15%, 25%) with protein intake in the two study phases.

Analyses shown with and without adjustment for clustering

Regression terms	Mean daily protein intake (kcal/day)	
	Unadjusted for clustering	Adjusted for clustering
	B ± se; P	B ± se; P
**Phase 1 (days 1-3)**		
Constant	131 ± 82; 0.11	178 ± 79; 0.03
Female sex (1=Y, 0=N)	-121 ± 28; <0.001	-121 ± 34; 0.001
Age (years)	3.4 ± 2.2; 0.12	1.5 ± 11.4; 0.43
Marasmus (1=Y, 0=N)	-20 ± 28; 0.28	-30 ± 24; 0.23
Body weight (kg)	1.6 ± 1.1; 0.16	1.3 ± 1.0; 0.19
Diet 10% (1=Y, 0=N)	32 ± 34; 0.35	44 ± 42; 0.30
Diet 25% (1=Y, 0=N)	-20 ± 34; 0.56	-16 ± 42; 0.71
**Phase 2 (days 4-8)**		
Constant	-142 ± 67, 0.04	-162 ± 61, 0.01
Female sex (1=Y, 0=N)	-158 ± 27; <0.001	-156 ± 33; 0.04
Age (years)	6.0 ± 2.1; 0.05	3.9 ± 1.9; 0.04
Marasmus (1=Y, 0=N)	1.9 ± 27.5; 0.94	-2.0 ± 23.5; 0.93
Body weight (kg)	2.6 ± 1.1; 0.02	2.7 ± 1.0; 0.01
Diet 10% (1=Y, 0=N)	-100 ± 34; 0.04	-92 ± 40.2; 0.03
Diet 25% (1=Y, 0=N)	147 ± 33; <0.001	151 ± 40.3; 0.001

B = regression coefficient; se = standard error; P = P-value.
