# Supplementary material for: Developmental contributions to macronutrient selection: a randomized controlled trial in adult survivors of malnutrition
Source: Evol Med Public Health. 2016 Jan 27;2016(1):158–69. doi: 10.1093/emph/eov030 (PMC4871598; doi:10.1093/emph/eov030)

Supplemental Data

Figure 4: Distribution of Energy Intake in 32 adult male survivors of severe acute malnutrition.

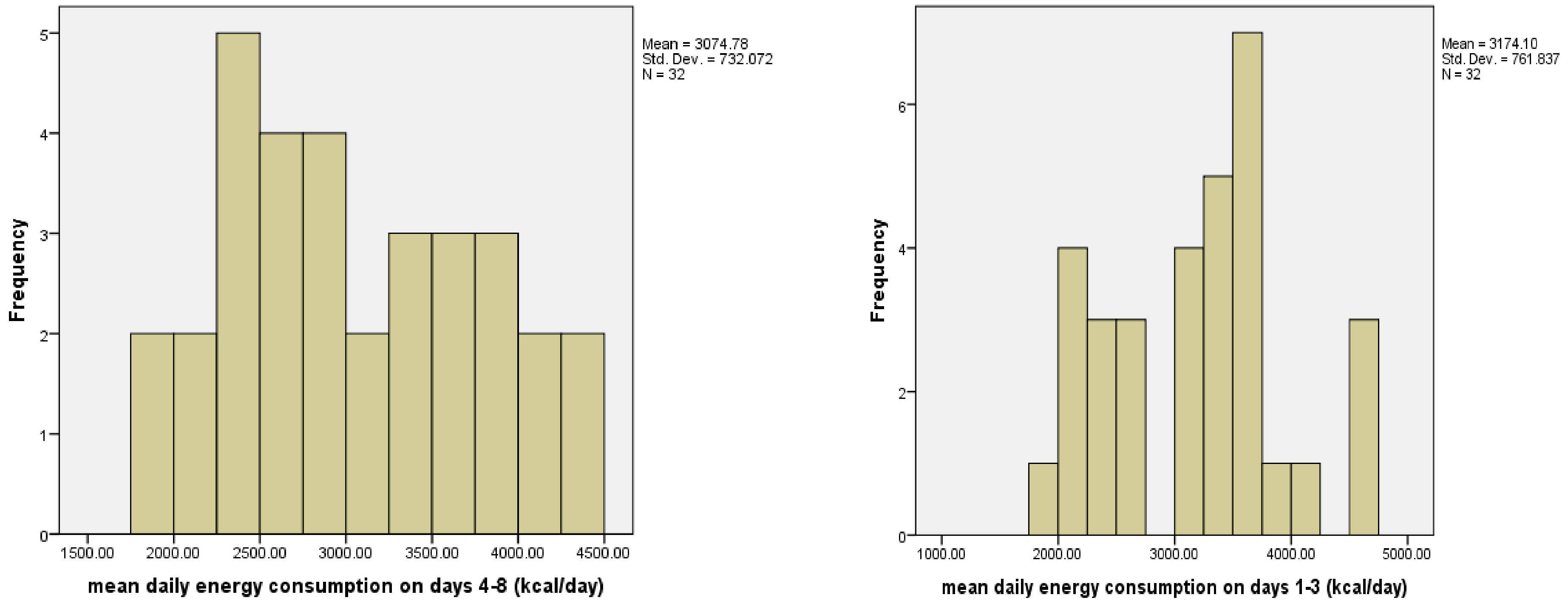

Figure 5: Distribution of Energy Intake in 31 adult female survivors of severe acute malnutrition.

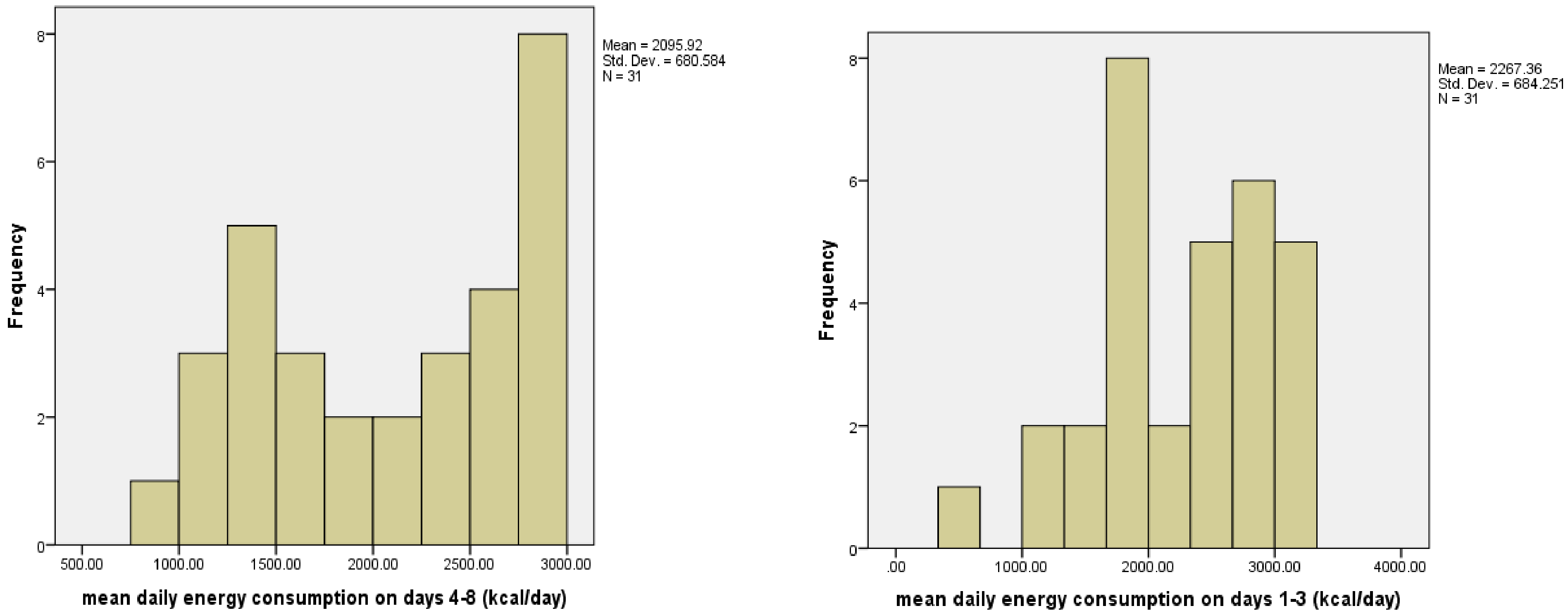

Supplement: Supplementary Data [file eov030_Supp.zip › Supplemental Data_Distribution of Energy ntake in Adult Survivors of Severe Acute Malnutrition.pdf]
